# Characterization of combined endoscopies and aerodigestive care: An analysis of utilization and financial feasibility

**DOI:** 10.1371/journal.pone.0291179

**Published:** 2023-09-06

**Authors:** Jennifer Brinkmeier, Noor Al-Hammadi, Sumana Shashidhar, Leslie Hinyard, Dhiren Patel

**Affiliations:** 1 Division of Pediatric Otolaryngology, Department of Otolaryngology, Washington University & Saint Louis Children’s Hospital, St. Louis, MO, United States of America; 2 Advanced HEAlth Data (AHEAD) Institute, Saint Louis University School of Medicine, St. Louis, MO, United States of America; 3 Department of Health and Clinical Outcomes Research, Saint Louis University, Saint Louis, MO, United States of America; 4 Saint Louis University School of Medicine, St. Louis, MO, United States of America; 5 Division of Pediatric Gastroenterology, Hepatology and Nutrition, Cardinal Glennon Children’s Medical Center, Saint Louis University School of Medicine, St. Louis, MO, United States of America; Kyung Hee University School of Medicine, REPUBLIC OF KOREA

## Abstract

**Background:**

Aerodigestive care is one model of multi-disciplinary care, which is a valuable tool for both providers and patients. Aerodigestive care models are associated with improved outcomes, reduced anesthesia exposure, reduction in hospital admissions, and fewer days of missed work or school. This is the first study to explore national usage and cost trends in combined endoscopy utilization to identify gaps in care and the potential for financial resource optimization.

**Methods:**

Data from the Healthcare Cost and Utilization Project (HCUP) Kid’s Inpatient Sample (KID) was used from 2016 and 2019. Diagnoses and procedures were identified using ICD-10 codes, for patients with hospital length of stay less than 1 day. Demographic data were identified, and survey-weighted means and proportions were computed. Bivariate comparisons were made using Rao Scott Chi-Square tests. National estimates of charges were computed with discharge weights, developed using the American Hospital Association (AHA) universe.

**Key results:**

White, high-income patients, and those at urban teaching hospitals received the greatest proportion of combined endoscopic procedures. The cost/charges associated with combined endoscopies are less than for separate gastrointestinal (GI) or airway only endoscopies combined. However, combined procedures comprise a smaller share of national aggregate cost.

**Conclusions:**

National utilization trends highlight racial and socioeconomic disparities and suggest differences in access based on hospital characteristics, despite the reduced cost/charges of the combined procedure. For patients with a need for combined aerodigestive procedures, there appears to be a cost-savings opportunity to increase efforts for combined procedures at the level of the clinician or hospital.

## Introduction

Multi-disciplinary care has become an increasingly valuable tool for providers, defined as all healthcare personnel involved in patient care during the hospital stay, and patients by allowing for improved coordination and communication, especially regarding complex patients [1, 2]. The structural organization of multi-disciplinary care inherently benefits patients by putting their needs at the forefront of the delivery model. Improved patient outcomes and satisfaction from care coordination has been repeatedly demonstrated as healthcare centers that implemented multi-disciplinary teams saw significant reductions in hospital days and admissions [1, 3–5], number of anesthetic exposures [6–8], and unnecessary testing [9–11]. The multi-disciplinary approach to care also benefits patients financially with reduced facility fees, parking and gas expenditures, and missed days of school or work [8, 12]. Furthermore, multi-disciplinary teams have positive financial implications for care centers. The improved efficiency afforded by multi-disciplinary teams and the same factors that benefit patients, such as reduced anesthetic exposures and length of hospital admissions, result in significant positive cost margins for many centers and insurance payers [1, 2, 8, 13–16].

Aerodigestive centers are an expanding form of multi-disciplinary care involving pulmonology, otolaryngology, gastroenterology, and services such as speech pathology. Within these centers, combined endoscopy is a key method of reducing anesthetic exposures as well as cost. Combined endoscopy involves any combination of procedures that fall under specialties involved in aerodigestive care (S1 and S2 Tables). The reduction in anesthetic episodes associated with combined endoscopy results in significant decreases to hospital charge and patient cost [8]. This combined procedure is also associated with improved targeted interventions and post-intervention health, for patients with and without diagnoses following the procedure [17]. Aerodigestive centers show similar benefits to other care coordination models for improved patient outcomes, patient satisfaction, cost, and innovation [1, 2, 8, 13–16].

Multi-disciplinary care has been increasingly adopted as a care model in recent times [18]; however, simply increasing the number of multi-disciplinary care centers does not directly correspond to an increase in access to these centers or procedures [11]. Considering the amount of money spent on airway and GI procedures alone, assessing the financial impact of combining procedures in addition to identifying gaps in care aids in understanding the role of combined procedures when there is a need. The purpose of this investigation is to examine the costs and charges as well as utilization trends based on socioeconomic factors for each procedure type in order to identify resource-saving opportunities and gaps in care for pediatric patients presenting with the need for airway and gastrointestinal endoscopy. The authors hypothesize that combined airway and GI procedures are both cost-effective and safer, when indicated for patient care.

## Materials and methods

### Data source, study sample and statistical considerations

The data were obtained from the Kid’s Inpatient Database (KID), Healthcare Cost and Utilization Project (HCUP), Agency for Healthcare Research and Quality [19], the largest publicly available all-payer database in the United States. The KID is released every three years. It contains about 3,000,000 annual pediatrics discharges with a rough estimate of 7 million weighted hospitalizations. This study used the 2016–2019 KID datasets and included procedures with a length of hospital stay of 1 day or less. Aerodigestive procedures were identified using ICD-10 procedure codes (S1 and S2 Tables). Specifically, airway procedures refer to the combined group of procedures that include laryngoscopy and bronchoscopy that may be performed by otolaryngology, pulmonology, or both, as the authors are familiar with broad variations across institutions. GI procedures refer primarily to procedures involving flexible esophagogastroduodenoscopy (EGD) performed by gastroenterology. In this study, combined procedures are defined as at least one GI procedure performed with an additional airway procedure as defined previously. Diagnoses associated with each procedure were identified using ICD-10 diagnosis codes and diagnoses representing complex aerodigestive patients were selected for further investigation (S3 Table). Procedures and diagnoses were reviewed by our senior clinician authors (JB DP) for relevance to the patient population. Patients were included in the study if they had any relevant aerodigestive procedure and one of the identified diagnoses. Patients were excluded if their length of hospital stay (length of stay, LOS) was greater than 1 day. Within the KID, procedure date is not specified, thus we restricted LOS to 1 day to ensure procedures occurred on same day and to limit the patient population to patients with a similar level of complexity. Patients were then categorized into three groups: Group 1) airway procedures alone, Group 2) GI procedures alone, and Group 3) both airway and GI procedures.

Hospital admission rates for the whole sample and within each treatment group were computed and analyzed by age, sex, race (White, Black, Hispanic, “other” and missing), insurance payer (governmental, private, other, and missing), median household income for patient’s ZIP code and year of the cohort. Hospital characteristics were also investigated. Weighted frequencies were calculated to represent national estimates for each procedure. The “other” category for race includes Asian or Pacific Islander, Native American, and other. The “other” category for insurance payer includes no charges and other. Patients were grouped into those receiving private insurance, governmental (Medicaid and Medicare) and “other” types. Total charges were inflation-adjusted to 2019 dollars using the Consumer Price Index (CPI) from the US Bureau of Labor Statistics [20]. The secondary outcome was total charges (hospital bill in then-US dollars). Charges and cost include the procedure itself as well as the associated hospital stay.

Survey-weighted means and proportions were computed for variables of interest in the whole sample and stratified by treatment option. Rao Scott Chi-Square [21] tests were used to compare the distribution of patients across the levels of each group. Discharge weights, developed using the American Hospital Association (AHA) universe, were used to provide the national estimates. Descriptive statistics were computed using SAS 9.4 (SAS Institute Inc., Cary, NC, USA). A two-sided P value less than 0.05 is considered significant for data in this study. The KID is de-identified and was thus deemed exempt by the IRB.

## Results

### Demographics

The study population sample size is 23,141 with a mean [SE] age of 4.07 [0.03] (18,274[0.33%] male; 13,191 [0.33%] female). This study population includes patients from the 2016 (16,238 [0.33%]) and 2019 (15,236 [0.33%]) waves, encompassing data from the years of 2013 through 2019 (Table 1). Of the total study population, a larger proportion identify as White (11,875 [0.32%]) than Hispanic (7,078 [28%]), or Black (6,576 [0.28%]). Moreover, 3,737 [0.22%] did not identify as either and 1,635 patients had missing inputs for race (Table 1). The largest group of patients in the study population fall under the highest income quartile (11,353 [0.31%]), with a decrease in number of patients as income percentile increases. The lowest income quartile had the smallest number of patients (5746 [0.25%]). Additionally, 17,919 [0.32%] of procedures had government payers while 8,233 [35.56%] of procedures had private payers and 1,695 [7.33%] had other forms of payer. Large hospitals and urban teaching hospitals dominate—14,814 [63.49%] of procedures studied took place at large hospitals; 20,401 [88.07%] of procedures took place in urban teaching hospitals (Table 1). Within the studied sample, most procedures took place in the Northeast (10,494 [0.17%]), with the fewest number of procedures taking place in the Midwest (4,046 [16.99%]) (Table 1).

**Table 1:** Patient demographics with baseline and hospital characteristics for children (<18 y) who received combined, airway only, or GI only endoscopies during hospital admission with LOS≤1 day; 2016 and 2019 cohorts.

	Total		Group 1: Airway only		Group 2: GI only		Group 3: Combined		p-value
	N (%)	Weighted Frequency (Std Err of %)	N (%)	Weighted Frequency (Std Err of %)	N (%)	Weighted Frequency (Std Err of %)	N (%)	Weighted Frequency (Std Err of %)	
**Sample**	23141 (100)	31474	16222 (69.97)	22024 (0.29)	6341 (27.52)	8663 (0.28)	578 (2.5)	788 (0.1)	** **
**Wave**									0.0006
2016 (2013 through 2016)	11850 (51.59)	16238 (0.33)	8467 (71.09)	11543 (0.41)	3108 (26.56)	4313 (0.4)	275 (2.35)	382 (0.14)	
2019 (2017 through 2019)	11291 (48.41)	15236 (0.33)	7755 (68.79)	10481 (0.42)	3233 (28.55)	4350 (0.41)	303 (2.66)	406 (0.15)	
**Baseline Characteristics**									
**Age (mean, Std Err)**	4.07 (0.03)	4.07 (0.03)	2.96 (0.03)	2.96 (0.03)	6.95 (0.07)	6.95 (0.07)	3.27 (0.19)	3.27 (0.19)	
**Race**									< .0001
1 (White)	8747 (40.59)	11875 (0.32)	5310 (60.58)	7193 (0.5)	3166 (36.32)	4312 (0.5)	271 (3.11)	369 (0.19)	
2 (Black)	4822 (22.48)	6576 (0.28)	3953 (81.99)	5391 (0.54)	782 (16.21)	1066 (0.52)	87 (1.8)	119 (0.19)	
3 (Hispanic)	5187 (24.19)	7078 (0.28)	3804 (73.19)	5180 (0.61)	1266 (24.56)	1739 (0.59)	117 (2.25)	159 (0.21)	
Other	2750 (12.74)	3727 (0.22)	2042 (74.15)	2763 (0.83)	645 (23.55)	878 (0.8)	63 (2.3)	86 (0.29)	
Missing	1635	1635							
**Gender**									< .0001
Male	13436 (58.08)	18274 (0.33)	9649 (71.7)	13102 (0.38)	3437 (25.7)	4696 (0.37)	350 (2.61)	477 (0.14)	
Female	9698 (41.92)	13191 (0.33)	6567 (67.58)	8914 (0.47)	2903 (30.07)	3966 (0.46)	228 (2.36)	311 (0.15)	
Missing	7	7							
**Income quartile**									< .0001
1 (0-25th percentile)	8311 (36.49)	11353 (0.31)	6636 (79.82)	9062 (0.43)	1539 (18.54)	2104 (0.42)	136 (1.64)	186 (0.14)	
2 (26th to 50th percentile (median)	5312 (23.26)	7236 (0.28)	3714 (69.87)	5055 (0.62)	1479 (27.89)	2018 (0.61)	119 (2.24)	162 (0.2)	
3 (51st to 75th percentile)	5007 (21.79)	6778 (0.27)	3196 (63.51)	4305 (0.68)	1639 (33.04)	2239 (0.66)	172 (3.45)	234 (0.26)	
4 (76th to 100th percentile)	4248 (18.47)	5746 (0.25)	2505 (58.59)	3366 (0.75)	1598 (37.97)	2182 (0.74)	145 (3.44)	198 (0.28)	
Missing	263	263							
**Payer**									< .0001
Governmental	13148 (57.1)	17919 (0.32)	9740 (73.97)	13254 (0.37)	3114 (23.79)	4263 (0.36)	294 (2.24)	401 (0.13)	
Private	8233 (35.56)	8233 (35.56)	5279 (63.94)	7135 (0.52)	2716 (33.16)	3700 (0.51)	238 (2.9)	324 (0.19)	
Other	1695 (7.33)	1695 (7.33)	1143 (67.19)	1546 (1.14)	507 (30.14)	694 (1.12)	45 (2.66)	61 (0.39)	
Missing	65	65							
**Hospital characteristics**									
**Bed Size**									< .0001
Small	3655 (16.1)	5069 (0.18)	2979 (81.48)	4130 (0.63)	629 (17.23)	873 (0.62)	47 (1.29)	65 (0.19)	
Medium	4672 (20.4)	4672 (20.4)	3374 (71.96)	4621 (0.61)	1203 (25.99)	1669 (0.6)	95 (2.05)	132 (0.21)	
Large	14814 (63.49)	14814 (63.49)	9869 (66.42)	13273 (0.37)	4509 (30.63)	6120 (0.36)	436 (2.96)	591 (0.14)	
**Hospital Region**									< .0001
Northeast	7702 (33.34)	10494 (0.17)	6645 (86.4)	9067 (0.38)	939 (12.08)	1268 (0.36)	118 (1.51)	159 (0.14)	
Midwest	4046 (16.99)	4046 (16.99)	2637 (64.72)	3461 (0.73)	1284 (32.15)	1719 (0.72)	125 (3.14)	168 (0.28)	
South	6177 (27.11)	6177 (27.11)	3925 (63.38)	5408 (0.58)	2111 (34.32)	2928 (0.57)	141 (2.3)	196 (0.19)	
West	5216 (22.56)	5216 (22.56)	3015 (57.58)	4088 (0.66)	2007 (38.69)	2747 (0.66)	194 (3.73)	265 (0.26)	
**Location/Teaching Status of Hospital**									< .0001
Rural	992 (4.58)	1442 (0.03)	932 (94.22)	1359 (0.72)	58 (5.6)	81 (0.71)	2 (0.18)	3 (0.13)	
Urban nonteaching	1748 (7.35)	1748 (7.35)	1476 (83.91)	1941 (0.85)	254 (15.05)	348 (0.83)	18 (1.04)	24 (0.24)	
Urban teaching	20401 (88.07)	20401 (88.07)	13814 (67.55)	18724 (0.31)	6029 (29.71)	8234 (0.31)	558 (2.75)	761 (0.11)	

### Procedure utilization

The study population was stratified by baseline and hospital characteristics, and the procedures associated with each subgroup—Group 1 airway only procedures, Group 2 GI only procedures, and Group 3 combined airway and GI procedures. Only 788 [0.1%] of patients studied received combined airway and GI procedures within the same admission. The proportion of each procedure type is consistent for both data waves, with a relatively balanced percentage of patients taken from each wave. When divided by race, each subgroup of patients was more likely to undergo airway only procedures. Overall, there were differences in the distributions of these procedures by patient race (p<0.0001). Black patients received the lowest number of combined procedures (119 [0.19%]) compared to White patients (369 [0.19%]), Hispanic patients (159 [0.21%]), and those in the “other” category (159 [0.21%]). Within the male study population, a greater number of airway only procedures (13,102 [0.38%]) were performed in comparison to females (p<0.0001). In contrast, females in this study population had a greater percentage of GI only procedures compared to males (p<0.0001). The number of combined procedures performed in males and females were at 477 [0.14%] and 311 [0.15%], respectively. Patients belonging to the lowest income quartile had the greatest proportion of airway procedures (9,062 [0.43%]), while patients in the top income quartiles had the greatest proportion of GI only procedures (2239 [0.66%], 2,182 [0.74%]) and combined procedures (234 [0.26%], 198 [0.28%]). In terms of insurance type, patients with governmental payers received the greatest proportion of combined procedures; however, the proportions significantly differ at 401 [0.13%] for governmental payer, 324 [0.19%] for private payer, and 61 [0.39%] for other types of insurance payers (p<0.0001). Airway only procedures comprised the greatest proportion of procedures conducted across all hospital characteristics groups. Among these hospital groups, large urban teaching hospitals (591 [0.14%] and 761 [0.11%], respectively) and hospitals in the West (265 [0.26%]) and South (196 [0.19%]) had the greatest proportion of combined procedures (Table 1).

### Charges and costs

**Fig 1:**
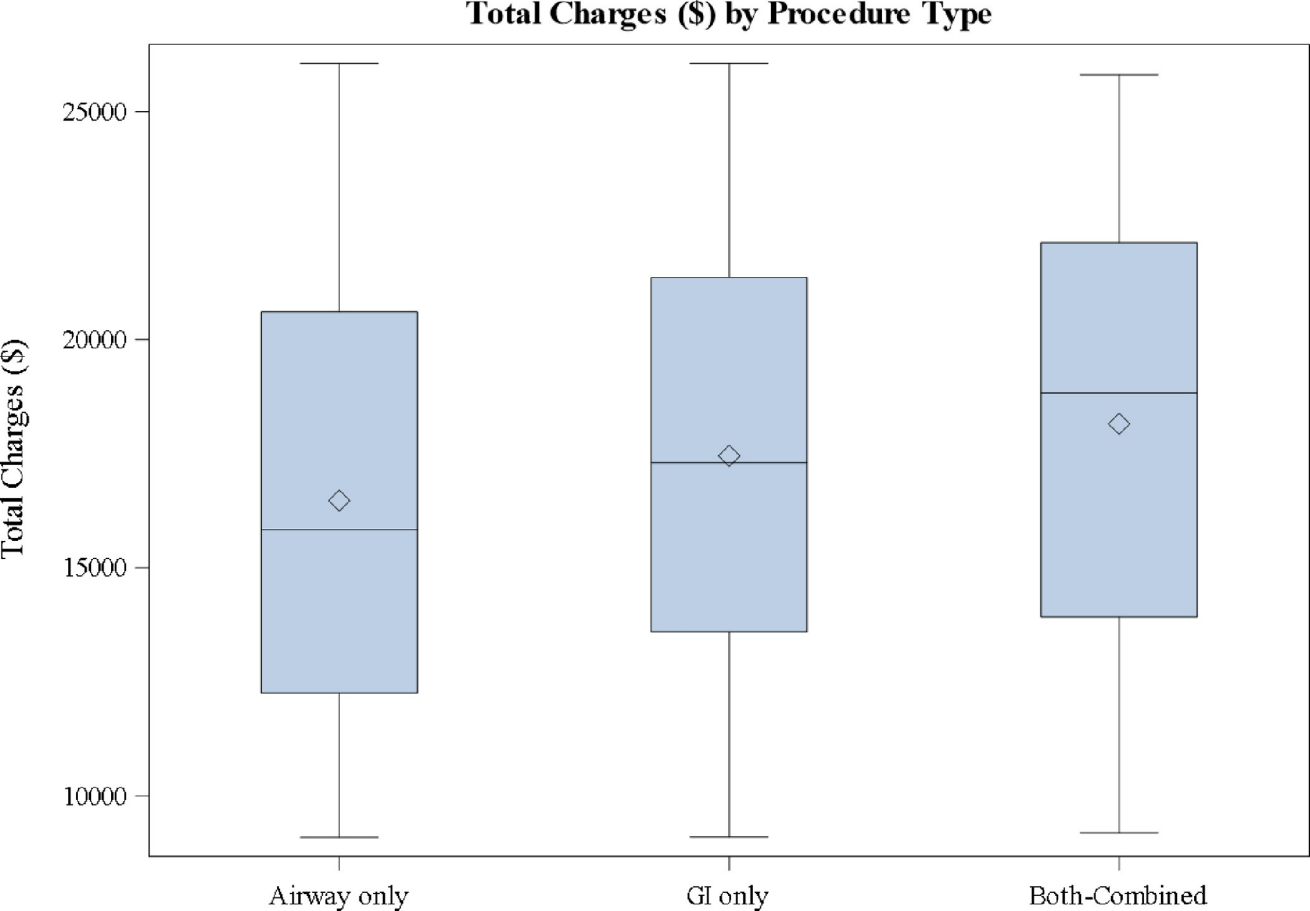
Total charges by procedure type; 2016 and 2019.

The national aggregate cost and therefore usage of combined airway and GI procedures (Group 3) is lower than for GI only or airway only procedures (Groups 1 and 2) (Table 3). The costs and charges associated with the combined procedure are significantly greater than the costs and charges of either independent procedure alone (p<0.0001) (Table 2). While the total mean charges associated with all three procedure categories was $16,838 (median $16,416), the mean charge of airway only procedures was $16,501 (median $15,874), the mean charge of GI only procedures was $17,472 (median $17,336), and the mean charge of combined procedures was $18,159 (median $18,836) (Table 2 and Fig 1). CMS standardized cost for the categories of procedures follows a similar pattern throughout. The total mean cost of the three procedure categories was $4,477 (median $4,099). When breaking down by procedure type, Table 2 shows that the mean cost of airway only procedures was $4,087 (median $3,618), the mean cost of GI only procedures was $5,215 (median $4,943), and the mean cost of combined procedures was $5,938 (median $5,749) (Table 2).

**Table 2:** Charges and costs by procedure type; 2016 and 2019.

	Total			Group 1: Airway only			Group 2: GI only			Group 3: Both			
	Mean ($)	Median ($)	n	Mean ($)	Median ($)	n	Mean ($)	Median ($)	n	Mean ($)	Median ($)	n	p-value
**Total Charges**	$16,838	$16,416	11398	$16,501	$15,874	7652	$17,472	$17,336	3479	$18,159	$18,836	267	< .0001
2016	$16,744	$16,287	5944	$16,422	$15,557	4038	$17,372	$17,184	1758	$17,927	$18,589	148	< .0001
2019	$16,943	$16,580	5454	$16,591	$16,114	3614	$17,576	$17,562	1721	$18,460	$19,310	119	< .0001
**Total Costs**	$4,477	$4,099	11398	$4,087	$3,618	7652	$5,215	$4,943	3479	$5,938	$5,749	267	< .0001
2016	$4,384	$3,977	5944	$3,960	$3,427	4038	$5,226	$4,896	1758	$5,789	$5,608	148	< .0001
2019	$4,580	$4,216	5454	$4,231	$3,806	3614	$5,204	$5,013	1721	$6,130	$6,211	119	< .0001

## Discussion

Children with special healthcare needs comprise about 15–18% of children in the United States but are responsible for more than 80% of health care costs for children [1, 22, 23]. Of the unscheduled admissions this population experiences, 32% are potentially preventable due to deficiencies in care coordination [1, 24]. Coordinated care in settings such as aerodigestive centers has repeatedly been shown to correspond to many benefits for patients, caregivers, and hospital systems. Along with improved satisfaction for patients and their caregivers, patients of these centers undergo fewer anesthetic procedures, experience reduced length of stay during hospital admissions, and have overall improved outcomes [25–27]. Benefits such as these must also be considered in the context of associated risks, such as increased early exposure to anesthesia and the proposed impact of neurotoxicity on the development of learning disabilities [25–27]. Additionally, the time and financial burden placed on caregivers is reduced with coordinated clinic appointments. Fewer missed days of school and work, and reduced spending on gas, parking, and facility fees contributes to family cost savings [8, 12]. With coordinated care, hospital systems not only gain financially due to reduced unnecessary testing [9–11] and shorter hospital stays, [1, 3–5] but also increase the potential for innovation with providers of different specialties working closely together [11]. With the guiding principle of coordinated care, and specifically coordinated procedures, increasingly emphasized, investigations of the components of coordinated care from national databases objectively inform how broadly adoption of coordinated care practices can be documented and evaluated via methodology complimentary to institutional and survey reporting. Ultimately, the confluence of these measures is aimed at delivering patient-centered care, reducing the clinical and healthcare-system burden on patients and families, and optimizing healthcare resources as possible.

To our knowledge, this is the first investigation to characterize national usage of aerodigestive procedures (GI, ENT, and pulmonary endoscopy) using a large public database. Although the benefits of multidisciplinary care coordination are clearly delineated in previous investigations, there is limited understanding of national usage and cost characteristics to examine where improvement can occur. Despite benefits to all stakeholders, national aggregate cost shows that there has not been a significant shift in how we deliver care for aerodigestive patients. From 2016 to 2019, the national aggregate cost of combined procedures and the associated hospital stay decreased by $457,874. However, combined procedures comprised an average of only $4,847,696 of the $191,628,566 total national cost of the three procedure groups with hospital stay over this time (Table 3). In contrast, airway only and GI only procedures with hospital stay comprise $126,058,696 and $60,722,175 of the national aggregate cost respectively. While this difference can partly be attributed to differences in need, the significant amount of money spent on these procedures indicates an opportunity to consider the financial opportunity of coordinated procedures and how they may represent a way to reduce unnecessary spending on multiple encounters with separate specialties when a combined case is indicated. The cost-savings opportunity afforded by combining procedures is evidenced by looking at the charges and costs associated with each procedure type. Although the charges and costs associated with the combined procedure is slightly higher than the GI only or airway only procedures, the cost of a combined procedures is significantly less than two separate procedures with their associated hospital stay. This highlights a potential for financial resource optimization with combined endoscopy; however, to improve utilization of combined procedures when indicated, we must first understand the current utilization trends.

**Table 3:** National aggregate cost by procedure type; 2016 and 2019.

	Total	Group 1: Airway only	Group 2: GI only	Group 3: Both
	Sum ($)	Sum ($)	Sum ($)	Sum ($)
**Aggregate Cost (National Bill)**	191,628,566	126,058,696	60,722,175	4,847,696
2016	99,173,998	66,023,360	30,497,854	2,652,785
2019	92,454,568	60,035,336	30,224,321	2,194,911

This study found that White pediatric patients comprise a majority of the study population receiving combined endoscopies. Overall, Black patients received the smallest number of total procedures, but the greatest proportion of airway only procedures compared to the other procedure types (Table 1). This is consistent with research that shows Black children having a higher prevalence of atopy associated conditions and requiring a greater proportion of airway only procedures [28, 29]. Patients in the lower income quartile received the greatest number of total procedures (8311 compared to 4248 procedures) and the smallest proportion of combined procedures (136 compared to 145 combined procedures). Income quartile is important to discuss when we consider the loss of work, need for additional transportation to a hospital, and potential for additional care for other family members that a second procedure might incur [12, 13]. The imperative to streamline care to a patient and family-centered model that prioritizes combined procedures when there is clinical need becomes quite clear. From here, we must consider if and how these racial and socioeconomic patterns arise from factors such as variations in access, clinical need, patient choice, provider decision-making, and financial security so that patient-centered improvements can be designed.

In terms of geographic access, combined endoscopies predominantly occurred at urban teaching hospitals with a greater number of these procedures taking place at large hospitals, specifically those in the Midwest and South (Table 1). In contrast, patients in rural areas receive substantially fewer combined procedures, potentially due to lack of access to care centers that can provide these services. This pattern elucidates that large, urban teaching hospitals have high use of combined procedures, though some of this is attributable to higher patient volume in those settings compared to other regions in this dataset. Additionally, hospitals in the Northeast had the greatest number of total procedures in comparison to other regions, but the smallest proportion of combined procedures. The utilization of combined procedures compared to airway or GI only procedures has not reached its maximum potential—the proportion of patients undergoing procedures in the same encounter is still quite low for what one would expect given the number or proportion of patients considered appropriate for multidisciplinary aerodigestive care [12, 13, 16].

One explanation for the low frequency of combined procedures is the numerous obstacles in the way of coordinated care. These obstacles include difficulty coordinating the schedules of multiple physicians and the patient, hospital resource allocation, and decision-making delays, among others as described by Golshan et al. regarding coordination between mastectomy and breast reconstructive surgery [30]. Although the number of aerodigestive programs has grown in recent years [31], combined endoscopies are subject to the same obstacles as other attempts at care coordination. As the authors of this paper have learned from experience practicing at an institution without an established aerodigestive care center, combined procedures are typically conducted largely due to the combined efforts of program coordinator personnel and clinicians. The low frequency may also represent that these procedures are increasingly performed in the outpatient setting over time. The benefits of combined procedures add to the urgency of improving the systemic and structural support, both from healthcare facilities and insurance providers, for aerodigestive care and multi-disciplinary collaboration. While some variation in utilization can be attributed to differences in patient need, coordinated care is patient-centered care, and clinicians’ efforts to improve it for patients that would benefit deserves institutional support.

The patterns this investigation has uncovered regarding geographic distribution and use of combined procedures exist partly due to the lack of coordinated care centers and the reliance upon the combined efforts of program coordinator personnel and clinicians. This study demonstrates that the charges and costs of combined procedures is consistently less than the sum of airway and GI procedures, which means clinically indicated combined procedures planned as coordinated care is a cost-savings opportunity. The organizational support within the infrastructure of coordinated care centers allows for more efficient and equitable use of combined procedures. We know that combining procedures is more cost effective [1, 2, 8, 13–16] and the data from this paper supports this. Therefore, improving coordinated care relies upon organizational support. This necessitates the need for assigning infrastructural support and revenue to establish programs such as aerodigestive centers and expand who benefits from coordinated care.

To design an intervention aimed at improving the use of and access to coordinated aerodigestive care, we must understand the current patterns of care, utilization, cost, and charges. Our data shows that combined procedures reduce the increased financial burden associated with two separate airway and GI procedures when clinically indicated. However, a larger number of centers may not directly correspond to expanded access to these procedures without addressing the underlying gaps in care. By characterizing the pediatric patients who require coordinated endoscopies, analyzing the patterns that exist, and investigating the financial viability of combining procedures, we can develop improvements in the aerodigestive care model that go beyond simply expanding in number.

There are limitations to this study that must be noted. First, there is a sizable amount of missing data on race due to changes in data collection and race categorization from 2016 through 2019 as well as differential reporting of information on race between included hospitals. Additionally, we utilized a proxy measure for combined aerodigestive procedures leading to the possibility of misclassification of procedures. However, misclassification is likely biased towards incorrectly assigning independent procedures as combined, thus the any bias in results likely dilutes the effect size. Lastly, this study is limited by the use of an inpatient database due to the limitation of outpatient databases in pediatrics.

## Conclusion

This investigation provides data on national trends associated with combined endoscopy procedures from the years 2013 through 2019. Understanding the current patterns of utilization, charges, and cost is necessary for improving equity across multi-disciplinary care models as they expand in number. Further research to evaluate outcomes with respect to their financial impact and to characterize disparities associated with combined procedures across settings with and without established aerodigestive centers will advance our ability to provide patient-centered care for these complex patients.

## Supporting information

S1 Table ICD-10 procedure codes used to identify airway endoscopies. (PDF)

S2 Table ICD-10 procedure codes used to identify GI endoscopies. (PDF)

S3 Table ICD-10 diagnosis codes used to identify complex aerodigestive patients. (PDF)
