# Supplementary material for: Characterization of combined endoscopies and aerodigestive care: An analysis of utilization and financial feasibility
Source: PLoS One. 2023 Sep 6;18(9):e0291179. doi: 10.1371/journal.pone.0291179 (PMC10482277; doi:10.1371/journal.pone.0291179)
Supplement: S1 Table — (PDF) [file pone.0291179.s001.pdf]

Table, Supplemental Digital Content 1 ICD-10 procedure codes used to identify airway endoscopies

ICD-10 procedure	Description
3E0.F3GC	Introduction of Other Therapeutic Substance into Respiratory Tract, Percutaneous Approach
3E0.F7GC	Introduction of Other Therapeutic Substance into Respiratory Tract, Via Natural or Artificial Opening
3E0.F8GC	Introduction of Other Therapeutic Substance into Respiratory Tract, Via Natural or Artificial Opening Endoscopic
0BJ.18ZZ	Inspection of Trachea, Via Natural or Artificial Opening Endoscopic
0BJ.14ZZ	Inspection of Trachea, Percutaneous Endoscopic Approach
0BJ.18ZZ	Inspection of Trachea, Via Natural or Artificial Opening Endoscopic
0CJ.S4ZZ	Inspection of Larynx, Percutaneous Endoscopic Approach
0CJ.S8ZZ	Inspection of Larynx, Via Natural or Artificial Opening Endoscopic
0C9.R7ZX	Drainage of Epiglottis, Via Natural or Artificial Opening, Diagnostic
0C9.R8ZX	Drainage of Epiglottis, Via Natural or Artificial Opening Endoscopic, Diagnostic
0C9.S7ZX	Drainage of Larynx, Via Natural or Artificial Opening, Diagnostic
0C9.S8ZX	Drainage of Larynx, Via Natural or Artificial Opening Endoscopic, Diagnostic
0C9.T7ZX	Drainage of Right Vocal Cord, Via Natural or Artificial Opening, Diagnostic
0C9.T8ZX	Drainage of Right Vocal Cord, Via Natural or Artificial Opening Endoscopic, Diagnostic
0C9.V7ZX	Drainage of Left Vocal Cord, Via Natural or Artificial Opening, Diagnostic
0C9.V8ZX	Drainage of Left Vocal Cord, Via Natural or Artificial Opening Endoscopic, Diagnostic
0CB.R7ZX	Excision of Epiglottis, Via Natural or Artificial Opening, Diagnostic
0CB.R8ZX	Excision of Epiglottis, Via Natural or Artificial Opening Endoscopic, Diagnostic

0CB.S7ZX	Excision of Larynx, Via Natural or Artificial Opening, Diagnostic
0CB.S8ZX	Excision of Larynx, Via Natural or Artificial Opening Endoscopic, Diagnostic
0CB.T7ZX	Excision of Right Vocal Cord, Via Natural or Artificial Opening, Diagnostic
0CB.T8ZX	Excision of Right Vocal Cord, Via Natural or Artificial Opening Endoscopic, Diagnostic
0CB.V7ZX	Excision of Left Vocal Cord, Via Natural or Artificial Opening, Diagnostic
0CB.V8ZX	Excision of Left Vocal Cord, Via Natural or Artificial Opening Endoscopic, Diagnostic
0B9.17ZX	Drainage of Trachea, Via Natural or Artificial Opening, Diagnostic
0B9.18ZX	Drainage of Trachea, Via Natural or Artificial Opening Endoscopic, Diagnostic
0B9.27ZX	Drainage of Carina, Via Natural or Artificial Opening, Diagnostic
0B9.28ZX	Drainage of Carina, Via Natural or Artificial Opening Endoscopic, Diagnostic
0CJ.S7ZZ	Inspection of Larynx, Via Natural or Artificial Opening
0BJ.17ZZ	Inspection of Trachea, Via Natural or Artificial Opening
0B5.13ZZ	Destruction of Trachea, Percutaneous Approach
0B5.14ZZ	Destruction of Trachea, Percutaneous Endoscopic Approach
0B5.17ZZ	Destruction of Trachea, Via Natural or Artificial Opening
0B5.18ZZ	Destruction of Trachea, Via Natural or Artificial Opening Endoscopic
0B5.23ZZ	Destruction of Carina, Percutaneous Approach
0B5.24ZZ	Destruction of Carina, Percutaneous Endoscopic Approach
0B5.27ZZ	Destruction of Carina, Via Natural or Artificial Opening
0B5.28ZZ	Destruction of Carina, Via Natural or Artificial Opening Endoscopic
0CQ.S4ZZ	Repair Larynx, Percutaneous Endoscopic Approach
0CQ.S7ZZ	Repair Larynx, Via Natural or Artificial Opening
0CQ.S8ZZ	Repair Larynx, Via Natural or Artificial Opening Endoscopic
0CQ.S4ZZ	Repair Larynx, Percutaneous Endoscopic Approach
0CQ.S7ZZ	Repair Larynx, Via Natural or Artificial Opening
0CQ.S8ZZ	Repair Larynx, Via Natural or Artificial Opening Endoscopic
0CQ.S3ZZ	Repair Larynx, Percutaneous Approach
0CQ.S4ZZ	Repair Larynx, Percutaneous Endoscopic Approach
0CQ.S7ZZ	Repair Larynx, Via Natural or Artificial Opening

0CQ.S8ZZ	Repair Larynx, Via Natural or Artificial Opening Endoscopic
0CQ.S4ZZ	Repair Larynx, Percutaneous Endoscopic Approach
0CQ.S7ZZ	Repair Larynx, Via Natural or Artificial Opening
0CQ.S8ZZ	Repair Larynx, Via Natural or Artificial Opening Endoscopic
0CQ.R4ZZ	Repair Epiglottis, Percutaneous Endoscopic Approach
0CQ.R7ZZ	Repair Epiglottis, Via Natural or Artificial Opening
0CQ.R8ZZ	Repair Epiglottis, Via Natural or Artificial Opening Endoscopic
0CQ.S3ZZ	Repair Larynx, Percutaneous Approach
0CQ.S4ZZ	Repair Larynx, Percutaneous Endoscopic Approach
0CQ.S7ZZ	Repair Larynx, Via Natural or Artificial Opening
0CQ.S8ZZ	Repair Larynx, Via Natural or Artificial Opening Endoscopic
0CQ.T3ZZ	Repair Right Vocal Cord, Percutaneous Approach
0CQ.T4ZZ	Repair Right Vocal Cord, Percutaneous Endoscopic Approach
0CQ.T7ZZ	Repair Right Vocal Cord, Via Natural or Artificial Opening
0CQ.T8ZZ	Repair Right Vocal Cord, Via Natural or Artificial Opening Endoscopic
0CQ.V3ZZ	Repair Left Vocal Cord, Percutaneous Approach
0CQ.V4ZZ	Repair Left Vocal Cord, Percutaneous Endoscopic Approach
0CQ.V7ZZ	Repair Left Vocal Cord, Via Natural or Artificial Opening
0CQ.V8ZZ	Repair Left Vocal Cord, Via Natural or Artificial Opening Endoscopic
0CR.S77Z	Replacement of Larynx with Autologous Tissue Substitute, Via Natural or Artificial Opening
0CR.S7JZ	Replacement of Larynx with Synthetic Substitute, Via Natural or Artificial Opening
0CR.S7KZ	Replacement of Larynx with Nonautologous Tissue Substitute, Via Natural or Artificial Opening
0CR.S87Z	Replacement of Larynx with Autologous Tissue Substitute, Via Natural or Artificial Opening Endoscopic
0CR.S8JZ	Replacement of Larynx with Synthetic Substitute, Via Natural or Artificial Opening Endoscopic

0CR.S8KZ	Replacement of Larynx with Nonautologous Tissue Substitute, Via Natural or Artificial Opening Endoscopic
0CR.T77Z	Replacement of Right Vocal Cord with Autologous Tissue Substitute, Via Natural or Artificial Opening
0CR.T7KZ	Replacement of Right Vocal Cord with Nonautologous Tissue Substitute, Via Natural or Artificial Opening
0CR.T87Z	Replacement of Right Vocal Cord with Autologous Tissue Substitute, Via Natural or Artificial Opening Endoscopic
0CR.T8KZ	Replacement of Right Vocal Cord with Nonautologous Tissue Substitute, Via Natural or Artificial Opening Endoscopic
0CR.V77Z	Replacement of Left Vocal Cord with Autologous Tissue Substitute, Via Natural or Artificial Opening
0CR.V7KZ	Replacement of Left Vocal Cord with Nonautologous Tissue Substitute, Via Natural or Artificial Opening
0CR.V8JZ	Replacement of Left Vocal Cord with Synthetic Substitute, Via Natural or Artificial Opening Endoscopic
0CR.V8KZ	Replacement of Left Vocal Cord with Nonautologous Tissue Substitute, Via Natural or Artificial Opening Endoscopic
0CS.T7ZZ	Reposition Right Vocal Cord, Via Natural or Artificial Opening
0CS.T8ZZ	Reposition Right Vocal Cord, Via Natural or Artificial Opening Endoscopic
0CS.V7ZZ	Reposition Left Vocal Cord, Via Natural or Artificial Opening
0CS.V8ZZ	Reposition Left Vocal Cord, Via Natural or Artificial Opening Endoscopic
0CU.S77Z	Supplement Larynx with Autologous Tissue Substitute, Via Natural or Artificial Opening
0CU.S7JZ	Supplement Larynx with Synthetic Substitute, Via Natural or Artificial Opening
0CU.S7KZ	Supplement Larynx with Nonautologous Tissue Substitute, Via Natural or Artificial Opening
0CU.S87Z	Supplement Larynx with Autologous Tissue Substitute, Via Natural or Artificial Opening Endoscopic
0CU.S8JZ	Supplement Larynx with Synthetic Substitute, Via Natural or Artificial Opening Endoscopic
0CU.S8KZ	Supplement Larynx with Nonautologous Tissue Substitute, Via Natural or Artificial Opening Endoscopic
0CU.T77Z	Supplement Right Vocal Cord with Autologous Tissue Substitute, Via Natural or Artificial Opening

0CU.T7JZ	Supplement Right Vocal Cord with Synthetic Substitute, Via Natural or Artificial Opening
0CU.T7KZ	Supplement Right Vocal Cord with Nonautologous Tissue Substitute, Via Natural or Artificial Opening
0CU.T87Z	Supplement Right Vocal Cord with Autologous Tissue Substitute, Via Natural or Artificial Opening Endoscopic
0CU.T8JZ	Supplement Right Vocal Cord with Synthetic Substitute, Via Natural or Artificial Opening Endoscopic
0CU.T8KZ	Supplement Right Vocal Cord with Nonautologous Tissue Substitute, Via Natural or Artificial Opening Endoscopic
0BQ.13ZZ	Repair Trachea, Percutaneous Approach
0BQ.14ZZ	Repair Trachea, Percutaneous Endoscopic Approach
0BQ.17ZZ	Repair Trachea, Via Natural or Artificial Opening
0BQ.18ZZ	Repair Trachea, Via Natural or Artificial Opening Endoscopic
0BQ.13ZZ	Repair Trachea, Percutaneous Approach
0BQ.14ZZ	Repair Trachea, Percutaneous Endoscopic Approach
0BQ.17ZZ	Repair Trachea, Via Natural or Artificial Opening
0BQ.18ZZ	Repair Trachea, Via Natural or Artificial Opening Endoscopic
0BQ.13ZZ	Repair Trachea, Percutaneous Approach
0BQ.14ZZ	Repair Trachea, Percutaneous Endoscopic Approach
0BQ.17ZZ	Repair Trachea, Via Natural or Artificial Opening
0BQ.18ZZ	Repair Trachea, Via Natural or Artificial Opening Endoscopic
0BW.13FZ	Revision of Tracheostomy Device in Trachea, Percutaneous Approach
0BW.14FZ	Revision of Tracheostomy Device in Trachea, Percutaneous Endoscopic Approach
0BN.13ZZ	Release Trachea, Percutaneous Approach
0BN.14ZZ	Release Trachea, Percutaneous Endoscopic Approach
0BN.17ZZ	Release Trachea, Via Natural or Artificial Opening
0BN.18ZZ	Release Trachea, Via Natural or Artificial Opening Endoscopic
0BN.23ZZ	Release Carina, Percutaneous Approach
0BN.24ZZ	Release Carina, Percutaneous Endoscopic Approach

0BN.27ZZ	Release Carina, Via Natural or Artificial Opening
0BN.28ZZ	Release Carina, Via Natural or Artificial Opening Endoscopic
0CN.R3ZZ	Release Epiglottis, Percutaneous Approach
0CN.R4ZZ	Release Epiglottis, Percutaneous Endoscopic Approach
0CN.R7ZZ	Release Epiglottis, Via Natural or Artificial Opening
0CN.R8ZZ	Release Epiglottis, Via Natural or Artificial Opening Endoscopic
0CN.S3ZZ	Release Larynx, Percutaneous Approach
0CN.S4ZZ	Release Larynx, Percutaneous Endoscopic Approach
0CN.S7ZZ	Release Larynx, Via Natural or Artificial Opening
0CN.S8ZZ	Release Larynx, Via Natural or Artificial Opening Endoscopic
0CN.T3ZZ	Release Right Vocal Cord, Percutaneous Approach
0CN.T4ZZ	Release Right Vocal Cord, Percutaneous Endoscopic Approach
0CN.T7ZZ	Release Right Vocal Cord, Via Natural or Artificial Opening
0CN.T8ZZ	Release Right Vocal Cord, Via Natural or Artificial Opening Endoscopic
0CN.V3ZZ	Release Left Vocal Cord, Percutaneous Approach
0CN.V4ZZ	2021 ICD-10-PCS 0CNV4ZZ Release Left Vocal Cord, Percutaneous Endoscopic Approach
0CN.V7ZZ	Release Left Vocal Cord, Via Natural or Artificial Opening
0CN.V8ZZ	Release Left Vocal Cord, Via Natural or Artificial Opening Endoscopic
0C7.S0DZ	Dilation of Larynx with Intraluminal Device, Open Approach
0C7.S3DZ	Dilation of Larynx with Intraluminal Device, Percutaneous Approach
0C7.S4DZ	Dilation of Larynx with Intraluminal Device, Percutaneous Endoscopic Approach
0C7.S7DZ	Dilation of Larynx with Intraluminal Device, Via Natural or Artificial Opening
0C7.S8DZ	Dilation of Larynx with Intraluminal Device, Via Natural or Artificial Opening Endoscopic
0CP.S3DZ	Removal of Intraluminal Device from Larynx, Percutaneous Approach
0CP.S7DZ	Removal of Intraluminal Device from Larynx, Via Natural or Artificial Opening
0CP.S8DZ	Removal of Intraluminal Device from Larynx, Via Natural or Artificial Opening Endoscopic
3E0.F3GC	Introduction of Other Therapeutic Substance into Respiratory Tract, Percutaneous Approach

3E0.F7GC	Introduction of Other Therapeutic Substance into Respiratory Tract, Via Natural or Artificial Opening
3E0.F8GC	Introduction of Other Therapeutic Substance into Respiratory Tract, Via Natural or Artificial Opening Endoscopic
0C7.S3ZZ	Dilation of Larynx, Percutaneous Approach
0C7.S4DZ	Dilation of Larynx with Intraluminal Device, Percutaneous Endoscopic Approach
0C7.S4ZZ	Dilation of Larynx, Percutaneous Endoscopic Approach
0C7.S7DZ	Dilation of Larynx with Intraluminal Device, Via Natural or Artificial Opening
0C7.S7ZZ	Dilation of Larynx, Via Natural or Artificial Opening
0C7.S8DZ	Dilation of Larynx with Intraluminal Device, Via Natural or Artificial Opening Endoscopic
0C7.S8ZZ	Dilation of Larynx, Via Natural or Artificial Opening Endoscopic
0CN.S3ZZ	Release Larynx, Percutaneous Approach
0CN.S4ZZ	Release Larynx, Percutaneous Endoscopic Approach
0CN.S7ZZ	Release Larynx, Via Natural or Artificial Opening
0CN.S8ZZ	Release Larynx, Via Natural or Artificial Opening Endoscopic
0CP.S3JZ	Removal of Synthetic Substitute from Larynx, Percutaneous Approach
0CP.S7JZ	Removal of Synthetic Substitute from Larynx, Via Natural or Artificial Opening
0CP.S8JZ	Removal of Synthetic Substitute from Larynx, Via Natural or Artificial Opening Endoscopic
0CR.T7JZ	Replacement of Right Vocal Cord with Synthetic Substitute, Via Natural or Artificial Opening
0CR.T8JZ	Replacement of Right Vocal Cord with Synthetic Substitute, Via Natural or Artificial Opening Endoscopic
0CR.V7JZ	Replacement of Left Vocal Cord with Synthetic Substitute, Via Natural or Artificial Opening
0CR.V87Z	Replacement of Left Vocal Cord with Autologous Tissue Substitute, Via Natural or Artificial Opening Endoscopic
0B7.13ZZ	Dilation of Trachea, Percutaneous Approach
0B7.14DZ	Dilation of Trachea with Intraluminal Device, Percutaneous Endoscopic Approach
0B7.14ZZ	Dilation of Trachea, Percutaneous Endoscopic Approach
0B7.17DZ	Dilation of Trachea with Intraluminal Device, Via Natural or Artificial Opening

0B7.17ZZ	Dilation of Trachea, Via Natural or Artificial Opening
0B7.18DZ	Dilation of Trachea with Intraluminal Device, Via Natural or Artificial Opening Endoscopic
0B7.18ZZ	Dilation of Trachea, Via Natural or Artificial Opening Endoscopic
0B7.23DZ	Dilation of Carina with Intraluminal Device, Percutaneous Approach
0B7.23ZZ	Dilation of Carina, Percutaneous Approach
0B7.24DZ	Dilation of Carina with Intraluminal Device, Percutaneous Endoscopic Approach
0B7.24ZZ	Dilation of Carina, Percutaneous Endoscopic Approach
0B7.27DZ	Dilation of Carina with Intraluminal Device, Via Natural or Artificial Opening
0B7.27ZZ	Dilation of Carina, Via Natural or Artificial Opening
0B7.28DZ	Dilation of Carina with Intraluminal Device, Via Natural or Artificial Opening Endoscopic
0B7.28ZZ	Dilation of Carina, Via Natural or Artificial Opening Endoscopic
0BQ.13ZZ	Repair Trachea, Percutaneous Approach
0BQ.14ZZ	Repair Trachea, Percutaneous Endoscopic Approach
0BQ.17ZZ	Repair Trachea, Via Natural or Artificial Opening
0BQ.18ZZ	Repair Trachea, Via Natural or Artificial Opening Endoscopic
0BQ.23ZZ	Repair Carina, Percutaneous Approach
0BQ.24ZZ	Repair Carina, Percutaneous Endoscopic Approach
0BQ.27ZZ	Repair Carina, Via Natural or Artificial Opening
0BQ.28ZZ	Repair Carina, Via Natural or Artificial Opening Endoscopic
0CH.Y3YZ	Insertion of Other Device into Mouth and Throat, Percutaneous Approach
0CH.Y7BZ	Insertion of Airway into Mouth and Throat, Via Natural or Artificial Opening
0CH.Y7YZ	Insertion of Other Device into Mouth and Throat, Via Natural or Artificial Opening
0CH.Y8BZ	Insertion of Airway into Mouth and Throat, Via Natural or Artificial Opening Endoscopic
0CH.Y8YZ	Insertion of Other Device into Mouth and Throat, Via Natural or Artificial Opening Endoscopic
0BJ.08ZZ	Inspection of Tracheobronchial Tree, Via Natural or Artificial Opening Endoscopic
0BJ.08ZZ	Inspection of Tracheobronchial Tree, Via Natural or Artificial Opening Endoscopic

0BJ.K8ZZ	Inspection of Right Lung, Via Natural or Artificial Opening Endoscopic
0BJ.L8ZZ	Inspection of Left Lung, Via Natural or Artificial Opening Endoscopic
0BJ.08ZZ	Inspection of Tracheobronchial Tree, Via Natural or Artificial Opening Endoscopic
0BJ.K8ZZ	Inspection of Right Lung, Via Natural or Artificial Opening Endoscopic
0BJ.L8ZZ	Inspection of Left Lung, Via Natural or Artificial Opening Endoscopic
0B9.33ZX	Drainage of Right Main Bronchus, Percutaneous Approach, Diagnostic
0B9.34ZX	Drainage of Right Main Bronchus, Percutaneous Endoscopic Approach, Diagnostic
0B9.37ZX	Drainage of Right Main Bronchus, Via Natural or Artificial Opening, Diagnostic
0B9.38ZX	Drainage of Right Main Bronchus, Via Natural or Artificial Opening Endoscopic, Diagnostic
0B9.43ZX	Drainage of Right Upper Lobe Bronchus, Percutaneous Approach, Diagnostic
0B9.44ZX	Drainage of Right Upper Lobe Bronchus, Percutaneous Endoscopic Approach, Diagnostic
0B9.47ZX	Drainage of Right Upper Lobe Bronchus, Via Natural or Artificial Opening, Diagnostic
0B9.48ZX	Drainage of Right Upper Lobe Bronchus, Via Natural or Artificial Opening Endoscopic, Diagnosti
0B9.53ZX	Drainage of Right Middle Lobe Bronchus, Percutaneous Approach, Diagnostic
0B9.54ZX	Drainage of Right Middle Lobe Bronchus, Percutaneous Endoscopic Approach, Diagnostic
0B9.57ZX	Drainage of Right Middle Lobe Bronchus, Via Natural or Artificial Opening, Diagnostic
0B9.58ZX	Drainage of Right Middle Lobe Bronchus, Via Natural or Artificial Opening Endoscopic, Diagnostic
0B9.63ZX	Drainage of Right Lower Lobe Bronchus, Percutaneous Approach, Diagnostic
0B9.64ZX	Drainage of Right Lower Lobe Bronchus, Percutaneous Endoscopic Approach, Diagnostic
0B9.67ZX	Drainage of Right Lower Lobe Bronchus, Via Natural or Artificial Opening, Diagnostic
0B9.68ZX	Drainage of Right Lower Lobe Bronchus, Via Natural or Artificial Opening Endoscopic, Diagnostic

0B9.73ZX	Drainage of Left Main Bronchus, Percutaneous Approach, Diagnostic
0B9.74ZX	Drainage of Left Main Bronchus, Percutaneous Endoscopic Approach, Diagnostic
0B9.77ZX	Drainage of Left Main Bronchus, Via Natural or Artificial Opening, Diagnostic
0B9.78ZX	Drainage of Left Main Bronchus, Via Natural or Artificial Opening Endoscopic, Diagnostic
0B983ZX	Drainage of Left Upper Lobe Bronchus, Percutaneous Approach, Diagnostic
0B9.84ZX	Drainage of Left Upper Lobe Bronchus, Percutaneous Endoscopic Approach, Diagnostic
0B9.87ZX	Drainage of Left Upper Lobe Bronchus, Via Natural or Artificial Opening, Diagnostic
0B9.88ZX	Drainage of Left Upper Lobe Bronchus, Via Natural or Artificial Opening Endoscopic, Diagnostic
0B9.93ZX	Drainage of Lingula Bronchus, Percutaneous Approach, Diagnostic
0B9.94ZX	Drainage of Lingula Bronchus, Percutaneous Endoscopic Approach, Diagnostic
0B9.97ZX	Drainage of Lingula Bronchus, Via Natural or Artificial Opening, Diagnostic
0B9.98ZX	Drainage of Lingula Bronchus, Via Natural or Artificial Opening Endoscopic, Diagnostic
0B9.B3ZX	Drainage of Left Lower Lobe Bronchus, Percutaneous Approach, Diagnostic
0B9.B4ZX	Drainage of Left Lower Lobe Bronchus, Percutaneous Endoscopic Approach, Diagnostic
0B9.B7ZX	Drainage of Left Lower Lobe Bronchus, Via Natural or Artificial Opening, Diagnostic
0B9.B8ZX	Drainage of Left Lower Lobe Bronchus, Via Natural or Artificial Opening Endoscopic, Diagnostic
0B9.K8ZX	Drainage of Right Lung, Via Natural or Artificial Opening Endoscopic, Diagnostic
0B9.L8ZX	Drainage of Left Lung, Via Natural or Artificial Opening Endoscopic, Diagnostic
0B9.M8ZX	Drainage of Bilateral Lungs, Via Natural or Artificial Opening Endoscopic, Diagnostic

4A0.985Z	Measurement of Respiratory Flow, Via Natural or Artificial Opening Endoscopic
0B7.33DZ	Dilation of Right Main Bronchus with Intraluminal Device, Percutaneous Approach
0B7.33ZZ	Dilation of Right Main Bronchus, Percutaneous Approach
0B7.34DZ	Dilation of Right Main Bronchus with Intraluminal Device, Percutaneous Endoscopic Approach
0B7.34ZZ	Dilation of Right Main Bronchus, Percutaneous Endoscopic Approach
0B7.37DZ	Dilation of Right Main Bronchus with Intraluminal Device, Via Natural or Artificial Opening
0B7.37ZZ	Dilation of Right Main Bronchus, Via Natural or Artificial Opening
0B7.38DZ	Dilation of Right Main Bronchus with Intraluminal Device, Via Natural or Artificial Opening Endoscopic
0B7.38ZZ	Dilation of Right Main Bronchus, Via Natural or Artificial Opening Endoscopic
0B7.43DZ	Dilation of Right Upper Lobe Bronchus with Intraluminal Device, Percutaneous Approach
0B7.43ZZ	Dilation of Right Upper Lobe Bronchus, Percutaneous Approach
0B7.44DZ	Dilation of Right Upper Lobe Bronchus with Intraluminal Device, Percutaneous Endoscopic Approach
0B7.44ZZ	Dilation of Right Upper Lobe Bronchus, Percutaneous Endoscopic Approach
0B7.47DZ	Dilation of Right Upper Lobe Bronchus with Intraluminal Device, Via Natural or Artificial Opening
0B7.47ZZ	Dilation of Right Upper Lobe Bronchus, Via Natural or Artificial Opening
0B7.48DZ	Dilation of Right Upper Lobe Bronchus with Intraluminal Device, Via Natural or Artificial Opening Endoscopic
0B7.48ZZ	Dilation of Right Upper Lobe Bronchus, Via Natural or Artificial Opening Endoscopic
0B7.53DZ	Dilation of Right Middle Lobe Bronchus with Intraluminal Device, Percutaneous Approach
0B7.53ZZ	Dilation of Right Middle Lobe Bronchus, Percutaneous Approach
0B7.54DZ	Dilation of Right Middle Lobe Bronchus with Intraluminal Device, Percutaneous Endoscopic Approach
0B7.54ZZ	Dilation of Right Middle Lobe Bronchus, Percutaneous Endoscopic Approach

0B7.57DZ	Dilation of Right Middle Lobe Bronchus with Intraluminal Device, Via Natural or Artificial Opening
0B7.57ZZ	Dilation of Right Middle Lobe Bronchus, Via Natural or Artificial Opening
0B7.58DZ	Dilation of Right Middle Lobe Bronchus with Intraluminal Device, Via Natural or Artificial Opening Endoscopic
0B7.63DZ	Dilation of Right Lower Lobe Bronchus with Intraluminal Device, Percutaneous Approach
0B7.63ZZ	Dilation of Right Lower Lobe Bronchus, Percutaneous Approach
0B7.64DZ	Dilation of Right Lower Lobe Bronchus with Intraluminal Device, Percutaneous Endoscopic Approach
0B7.64ZZ	Dilation of Right Lower Lobe Bronchus, Percutaneous Endoscopic Approach
0B7.67DZ	Dilation of Right Lower Lobe Bronchus with Intraluminal Device, Via Natural or Artificial Opening
0B7.67ZZ	Dilation of Right Lower Lobe Bronchus, Via Natural or Artificial Opening
0B7.68DZ	Dilation of Right Lower Lobe Bronchus with Intraluminal Device, Via Natural or Artificial Opening Endoscopic
0B7.68ZZ	Dilation of Right Lower Lobe Bronchus, Via Natural or Artificial Opening Endoscopic
0B7.73DZ	Dilation of Left Main Bronchus with Intraluminal Device, Percutaneous Approach
0B7.73ZZ	Dilation of Left Main Bronchus, Percutaneous Approach
0B7.74DZ	Dilation of Left Main Bronchus with Intraluminal Device, Percutaneous Endoscopic Approach
0B7.74ZZ	Dilation of Left Main Bronchus, Percutaneous Endoscopic Approach
0B7.77DZ	Dilation of Left Main Bronchus with Intraluminal Device, Via Natural or Artificial Opening
0B7.77ZZ	Dilation of Left Main Bronchus, Via Natural or Artificial Opening
0B7.78DZ	Dilation of Left Main Bronchus with Intraluminal Device, Via Natural or Artificial Opening Endoscopic
0B7.78ZZ	Dilation of Left Main Bronchus, Via Natural or Artificial Opening Endoscopic
0B7.83DZ	Dilation of Left Upper Lobe Bronchus with Intraluminal Device, Percutaneous Approach
0B7.83ZZ	Dilation of Left Upper Lobe Bronchus, Percutaneous Approach
0B7.84DZ	Dilation of Left Upper Lobe Bronchus with Intraluminal Device, Percutaneous Endoscopic Approach

0B7.84ZZ	Dilation of Left Upper Lobe Bronchus, Percutaneous Endoscopic Approach
0B7.87DZ	Dilation of Left Upper Lobe Bronchus with Intraluminal Device, Via Natural or Artificial Opening
0B7.87ZZ	Dilation of Left Upper Lobe Bronchus, Via Natural or Artificial Opening
0B7.88DZ	Dilation of Left Upper Lobe Bronchus with Intraluminal Device, Via Natural or Artificial Opening Endoscopic
0B7.88ZZ	Dilation of Left Upper Lobe Bronchus, Via Natural or Artificial Opening Endoscopic
0B7.93DZ	Dilation of Lingula Bronchus with Intraluminal Device, Percutaneous Approach
0B7.93ZZ	Dilation of Lingula Bronchus, Percutaneous Approach
0B7.94DZ	Dilation of Lingula Bronchus with Intraluminal Device, Percutaneous Endoscopic Approach
0B7.94ZZ	Dilation of Lingula Bronchus, Percutaneous Endoscopic Approach
0B7.97DZ	Dilation of Lingula Bronchus with Intraluminal Device, Via Natural or Artificial Opening
0B7.97ZZ	Dilation of Lingula Bronchus, Via Natural or Artificial Opening
0B7.98DZ	Dilation of Lingula Bronchus with Intraluminal Device, Via Natural or Artificial Opening Endoscopic
0B7.98ZZ	Dilation of Lingula Bronchus, Via Natural or Artificial Opening Endoscopic
0B7.B7DZ	Dilation of Left Lower Lobe Bronchus with Intraluminal Device, Via Natural or Artificial Opening
0B7.B7ZZ	Dilation of Left Lower Lobe Bronchus, Via Natural or Artificial Opening
0B7.B8DZ	Dilation of Left Lower Lobe Bronchus with Intraluminal Device, Via Natural or Artificial Opening Endoscopic
0B7.B8ZZ	Dilation of Left Lower Lobe Bronchus, Via Natural or Artificial Opening Endoscopic
