# Supplementary material for: Characterization of combined endoscopies and aerodigestive care: An analysis of utilization and financial feasibility
Source: PLoS One. 2023 Sep 6;18(9):e0291179. doi: 10.1371/journal.pone.0291179 (PMC10482277; doi:10.1371/journal.pone.0291179)
Supplement: S2 Table — (PDF) [file pone.0291179.s002.pdf]

Table, Supplemental Digital Content 2 ICD-10 procedure codes used to identify GI endoscopies

ICD-10 procedure	Description
0DJ.08ZZ	Inspection of Upper Intestinal Tract, Via Natural or Artificial Opening Endoscopic
0D9.58ZX	Drainage of Esophagus, Via Natural or Artificial Opening Endoscopic, Diagnostic
0DJ.07ZZ	Inspection of Upper Intestinal Tract, Via Natural or Artificial Opening
0D5.58ZZ	Destruction of Esophagus, Via Natural or Artificial Opening Endoscopic
0W3.P8ZZ	Control Bleeding in Gastrointestinal Tract, Via Natural or Artificial Opening Endoscopic
3E0.G8TZ	Introduction of Destructive Agent into Upper GI, Via Natural or Artificial Opening Endoscopic
0D5.57ZZ	Destruction of Esophagus, Via Natural or Artificial Opening
0DH.54DZ	Insertion of Intraluminal Device into Esophagus, Percutaneous Endoscopic Approach
0DH.54UZ	Insertion of Feeding Device into Esophagus, Percutaneous Endoscopic Approach
0DH.57DZ	Insertion of Intraluminal Device into Esophagus, Via Natural or Artificial Opening
0DH.57UZ	Insertion of Feeding Device into Esophagus, Via Natural or Artificial Opening
0DH.58DZ	Insertion of Intraluminal Device into Esophagus, Via Natural or Artificial Opening Endoscopic
0DH.58UZ	Insertion of Feeding Device into Esophagus, Via Natural or Artificial Opening Endoscopic
0D7.44DZ	Dilation of Esophagogastric Junction with Intraluminal Device, Percutaneous Endoscopic Approach
0D7.54DZ	Dilation of Esophagus with Intraluminal Device, Percutaneous Endoscopic Approach
06L.34ZZ	Occlusion of Esophageal Vein, Percutaneous Endoscopic Approach
0D7.17DZ	Dilation of Upper Esophagus with Intraluminal Device, Via Natural or Artificial Opening
0D7.17ZZ	Dilation of Upper Esophagus, Via Natural or Artificial Opening
0D7.18DZ	Dilation of Upper Esophagus with Intraluminal Device, Via Natural or Artificial Opening Endoscopic

0D7.18ZZ	Dilation of Upper Esophagus, Via Natural or Artificial Opening Endoscopic
0D7.27DZ	Dilation of Middle Esophagus with Intraluminal Device, Via Natural or Artificial Opening
0D7.27ZZ	Dilation of Middle Esophagus, Via Natural or Artificial Opening
0D7.28DZ	Dilation of Middle Esophagus with Intraluminal Device, Via Natural or Artificial Opening Endoscopic
0D7.28ZZ	Dilation of Middle Esophagus, Via Natural or Artificial Opening Endoscopic
0D7.37DZ	Dilation of Lower Esophagus with Intraluminal Device, Via Natural or Artificial Opening
0D7.37ZZ	Dilation of Lower Esophagus, Via Natural or Artificial Opening
0D7.38DZ	Dilation of Lower Esophagus with Intraluminal Device, Via Natural or Artificial Opening Endoscopic
0D7.38ZZ	Dilation of Lower Esophagus, Via Natural or Artificial Opening Endoscopic
0D7.47DZ	Dilation of Esophagogastric Junction with Intraluminal Device, Via Natural or Artificial Opening
0D7.47ZZ	Dilation of Esophagogastric Junction, Via Natural or Artificial Opening
0D7.48DZ	Dilation of Esophagogastric Junction with Intraluminal Device, Via Natural or Artificial Opening Endoscopic
0D7.48ZZ	Dilation of Esophagogastric Junction, Via Natural or Artificial Opening Endoscopic
0D7.57DZ	Dilation of Esophagus with Intraluminal Device, Via Natural or Artificial Opening
0D7.57ZZ	Dilation of Esophagus, Via Natural or Artificial Opening
0D7.58DZ	Dilation of Esophagus with Intraluminal Device, Via Natural or Artificial Opening Endoscopic
0D7.58ZZ	Dilation of Esophagus, Via Natural or Artificial Opening Endoscopic
0D.H63UZ	Insertion of Feeding Device into Stomach, Percutaneous Approach
0DH.64UZ	Insertion of Feeding Device into Stomach, Percutaneous Endoscopic Approach
0DH.60UZ	Insertion of Feeding Device into Stomach, Open Approach
0D5.67ZZ	Destruction of Stomach, Via Natural or Artificial Opening
0D5.68ZZ	Destruction of Stomach, Via Natural or Artificial Opening Endoscopic
0D5.64ZZ	Destruction of Stomach, Percutaneous Endoscopic Approach
0DJ.64ZZ	Inspection of Stomach, Percutaneous Endoscopic Approach
0DJ.68ZZ	Inspection of Stomach, Via Natural or Artificial Opening Endoscopic

0DJ.68ZZ	Inspection of Stomach, Via Natural or Artificial Opening Endoscopic
0D9.63ZX	Drainage of Stomach, Via Natural or Artificial Opening, Diagnostic
0D9.64ZX	Drainage of Stomach, Percutaneous Endoscopic Approach, Diagnostic
0D9.67ZX	Drainage of Stomach, Via Natural or Artificial Opening, Diagnostic
0D9.68ZX	Drainage of Stomach, Via Natural or Artificial Opening Endoscopic, Diagnostic
0DB.64ZX	Excision of Stomach, Percutaneous Endoscopic Approach, Diagnostic
0DB.63ZX	Excision of Stomach, Percutaneous Approach, Diagnostic
0DJ.67ZZ	Inspection of Stomach, Via Natural or Artificial Opening
0D7.74DZ	Dilation of Stomach, Pylorus with Intraluminal Device, Percutaneous Endoscopic Approach
0D7.78DZ	Dilation of Stomach, Pylorus with Intraluminal Device, Via Natural or Artificial Opening Endoscopic
0D7.78ZZ	Dilation of Stomach, Pylorus, Via Natural or Artificial Opening Endoscopic
0DW.08UZ	Revision of Feeding Device in Upper Intestinal Tract, Via Natural or Artificial Opening Endoscopic
0DW.04UZ	Revision of Feeding Device in Upper Intestinal Tract, Percutaneous Endoscopic Approach
0DQ.68ZZ	Repair Stomach, Via Natural or Artificial Opening Endoscopic
0DQ.67ZZ	Repair Stomach, Via Natural or Artificial Opening
0DQ.94ZZ	Repair Duodenum, Percutaneous Endoscopic Approach
0DQ.98ZZ	Repair Duodenum, Via Natural or Artificial Opening Endoscopic
0DQ.64ZZ	Repair Stomach, Percutaneous Endoscopic Approach
0DQ.68ZZ	Repair Stomach, Via Natural or Artificial Opening Endoscopic
0DQ.98ZZ	Repair Duodenum, Via Natural or Artificial Opening Endoscopic
0DQ.94ZZ	Repair Duodenum, Percutaneous Endoscopic Approach
0W3.P8ZZ	Control Bleeding in Gastrointestinal Tract, Via Natural or Artificial Opening Endoscopic
0DQ.98ZZ	Repair Duodenum, Via Natural or Artificial Opening Endoscopic
0DQ.94ZZ	Repair Duodenum, Percutaneous Endoscopic Approach
0DQ.68ZZ	Repair Stomach, Via Natural or Artificial Opening Endoscopic

0DQ.64ZZ	Repair Stomach, Percutaneous Endoscopic Approach
0DJ.D4ZZ	Inspection of Lower Intestinal Tract, Percutaneous Endoscopic Approach
0DJ.07ZZ	Endoscopy of small intestine through artificial stoma
0DJ.08ZZ	Inspection of Upper Intestinal Tract, Via Natural or Artificial Opening Endoscopic
0D9.83ZX	Drainage of Small Intestine, Percutaneous Approach, Diagnostic
0D9.84ZX	Drainage of Small Intestine, Percutaneous Endoscopic Approach, Diagnostic
0D9.87ZX	Drainage of Small Intestine, Via Natural or Artificial Opening, Diagnostic
0D9.93ZX	Drainage of Duodenum, Percutaneous Approach, Diagnostic
0D9.94ZX	Drainage of Duodenum, Percutaneous Endoscopic Approach, Diagnostic
0D9.97ZX	Drainage of Duodenum, Via Natural or Artificial Opening, Diagnostic
0D9.58ZX	Drainage of Esophagus, Via Natural or Artificial Opening Endoscopic, Diagnostic
0D9.68ZX	Drainage of Stomach, Via Natural or Artificial Opening Endoscopic, Diagnostic
0D9.98ZX	Drainage of Duodenum, Via Natural or Artificial Opening Endoscopic, Diagnostic
0DB.58ZX	Excision of Esophagus, Via Natural or Artificial Opening Endoscopic, Diagnostic
0DB.68ZX	Excision of Stomach, Via Natural or Artificial Opening Endoscopic, Diagnostic
0DB.98ZX	Excision of Duodenum, Via Natural or Artificial Opening Endoscopic, Diagnostic
0DJ.07ZZ	Inspection of Upper Intestinal Tract, Via Natural or Artificial Opening
0DJ.03ZZ	Inspection of Upper Intestinal Tract, Percutaneous Approach
0DB.88ZZ	Excision of Small Intestine, Via Natural or Artificial Opening Endoscopic
0DB.87ZZ	Excision of Small Intestine, Via Natural or Artificial Opening

0DH.A8UZ	Insertion of Feeding Device into Jejunum, Via Natural or Artificial Opening Endoscopic
0DH.A4UZ	Insertion of Feeding Device into Jejunum, Percutaneous Endoscopic Approach
0DH.A3UZ	Insertion of Feeding Device into Jejunum, Percutaneous Approach
0DC.58ZZ	Extirpation of Matter from Esophagus, Via Natural or Artificial Opening Endoscopic
0DC.57ZZ	Extirpation of Matter from Esophagus, Via Natural or Artificial Opening
0DC.67ZZ	Extirpation of Matter from Stomach, Via Natural or Artificial Opening
0DC.68ZZ	Extirpation of Matter from Stomach, Via Natural or Artificial Opening Endoscopic
0DC.87ZZ	Extirpation of Matter from Small Intestine, Via Natural or Artificial Opening
0D2.0X0Z	Change Drainage Device in Upper Intestinal Tract, External Approach
0D2.0XUZ	Change Feeding Device in Upper Intestinal Tract, External Approach
0D2.0XYZ	Change Other Device in Upper Intestinal Tract, External Approach
0D2.0X0Z	Change Drainage Device in Upper Intestinal Tract, External Approach
0D2.0XUZ	Change Feeding Device in Upper Intestinal Tract, External Approach
0D2.0XYZ	Change Other Device in Upper Intestinal Tract, External Approach
0DP.0XUZ	Removal of Feeding Device from Upper Intestinal Tract, External Approach
0DP.0X0Z	Removal of Drainage Device from Upper Intestinal Tract, External Approach
