# Supplementary material for: Characterization of combined endoscopies and aerodigestive care: An analysis of utilization and financial feasibility
Source: PLoS One. 2023 Sep 6;18(9):e0291179. doi: 10.1371/journal.pone.0291179 (PMC10482277; doi:10.1371/journal.pone.0291179)
Supplement: S3 Table — (PDF) [file pone.0291179.s003.pdf]

Table, Supplemental Digital Content 3 ICD-10 diagnosis codes used to identify complex aerodigestive patients

**Condition**	**ICD-10 Dx Code**	**N**	**%**
ESOPHAGEAL REFLUX	'53081','K219'	2704	11.7696
FEEDING PROBLEM	'7833','R633'	1399	6.0961
GASTROSTOMY STATUS	'V441','Z931'	1319	5.7512
FAILURE TO THRIVE	'78341','R6251'	1244	5.4006
DYSPHAGIA	'78720','R1310'	1194	5.1832
LACK NORM PHYSIOL DEVELOP	'78340','R6250'	913	3.9822
TRACHEA & BRONCH DIS NEC	51919','5191','J398','J9801','J9809'	779	3.3862
ESOPHAGITIS	53010','K209','K209','K210','K209','K200','K208','530'	633	2.7485
STENOSIS OF LARYNX	'78720','R1310','7872','R1310','R1311','R1312','R1313','R1314'	633	2.7459
STENOSIS OF LARYNX	'47874','J386'	633	2.7459
TRACHEOSTOMY STATUS	'V440 ','Z930'	585	2.5464
STRIDOR	'7861','R061'	492	2.1376
CROUP	'4644','J050'	444	1.9183
PERINATAL CHR RESP DIS	'7707','P271','P278'	443	1.9175
RESPIRATOR DEPEND STATUS	'V4611','Z9911'	427	1.8615
GSTR/DDNTS NOS W/O HMRHG	'53550','K2970','K2990'	343	1.4919
TRACHEOST COMPL	'51909','J9501','J9503','J9504','J9509'	323	1.4112
RESPIRATORY ABNORM NEC	'78609 ','R0600','R0609','R063','R0689'	322	1.3992
RESPIRATORY ABNORM NEC	'78609','R0600','R0609','R063','R0689'	322	1.3992
ATTEN TO GASTROSTOMY	'V551','Z431'	287	1.2424
CEREBRAL PALSY NOS	'3439','G809'	282	1.2263
GASTROST MECH COMPL	'53642','K9423'	273	1.1881
HEMATEMESIS	'5780','K920'	252	1.0986
HEMATEMESIS	'5780','K920'	252	1.0986
ESOPHAGEAL STRICTURE	'5303','K222'	251	1.0876
ESOPHAGEAL STRICTURE	'5303','K222'	251	1.0876
ATTEN TO TRACHEOSTOMY	'V550','Z430'	235	1.0231
COUGH	'7862','R05'	226	0.9792
DISEASE OF LARYNX NEC	'47879','J387'	165	0.7189
SPECFIED CONG ANOMAL NEC	'75989','Q8789','Q898'	140	0.6146
FOREIGN BODY ESOPHAGUS	'T18100A','T18100D','T18100S','T18108A','T18108D','T18108S'	64	0.2785
OTH SPF GSTRT W/O HMRHG	'53540','K2960','K2961'	60	0.259

OTHER SPEC SUSPECTED CONDITIONS	'V7189','Z036','Z0389'	17	0.0732
BRONCHITIS NOS	'J40 ','490'	11	0.0473
LARYNGOTRACH ANOMALY NEC	'7483','Q31'	0	0
